# Can Wolf Predation Immediately Alter the Foraging Behavior of Beavers?: Video of a Wolf Killing a Foraging Beaver

**DOI:** 10.1002/ece3.71357

**Published:** 2025-05-10

**Authors:** Danielle R. Freund, Thomas D. Gable, Austin T. Homkes, Olivia R. Jensen, Sage Patchett, Joseph K. Bump

**Affiliations:** ^1^ Department of Fisheries, Wildlife, and Conservation Biology University of Minnesota St Paul Minnesota USA; ^2^ Department of Environmental and Life Sciences Trent University Peterborough Ontario Canada

**Keywords:** alternate prey, anti‐predator behavior, predation risk, predator–prey, predatory behavior

## Abstract

Knowledge of wolf ( *Canis lupus* )‐beaver ( *Castor canadensis* ) interactions has largely been derived from indirect observations due to the cryptic nature of wolves and the densely forested areas where they usually kill beavers. In September 2023, we captured a video via remote camera of a wolf killing an adult beaver that was foraging on a feeding trail. This observation provides insight into how wolves may prevent beavers from reaching water after an attack is initiated, as well as how beavers attempt to escape once attacked. The camera also recorded the number of beavers foraging before and after the kill, providing a unique opportunity to observe the foraging behavior of the surviving beavers. The camera recorded videos on the trail for 11 nights before the predation and 37 nights after the predation. The time beavers spent on the feeding trail declined by 96% following predation. Although we present just a single observation, it raises an interesting question: is it possible or even plausible to think wolves might immediately alter where or the extent to which beavers forage through predation? We provide a detailed discussion on possibilities and highlight areas for future research.

## Graphical abstract

Knowledge of wolf‐beaver interactions has largely been derived from indirect observations due to the cryptic nature of wolves and the densely forested areas where they regularly kill beavers. In September 2023, we opportunistically recorded a video on a remote camera of a wolf killing an adult beaver, providing direct evidence of how wolves kill beavers. Our data provide unique insight on difficult‐to‐observe predatory behavior.

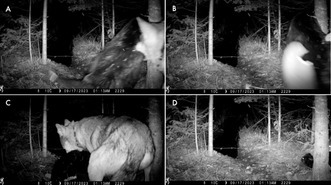

## 1 Introduction

Wolves are the primary natural predators of beavers wherever they co‐occur (Gable et al. 2016) with beavers constituting up to 42% of wolf pack diets during spring and fall in northern Minnesota (Gable and Windels 2018). Yet, observing wolf‐beaver interactions is challenging due to the cryptic nature of wolves and the densely vegetated landscapes where they typically hunt beavers (Gable et al. 2016). Because beavers are crepuscular/nocturnal, they predominantly forage on land at night where they are at a higher risk of predation (Novak et al. 1987; Rosell et al. 2006; Rosell and Campbell‐Palmer 2022). Wolf‐beaver interactions are also difficult to observe because wolves often use ambush strategies to kill beavers (Gable et al. 2016, 2021), and are able to kill and consume a beaver within a few hours (Gable et al. 2023; Peterson and Ciucci 2003).

Accordingly, the majority of research investigating wolf predation on beavers has occurred through indirect observations (Gable et al. 2016, 2020, 2021, 2023; Gable and Windels 2018), due to the difficulty of directly observing how wolves kill beavers (e.g., eye‐witness or videos). Previous research using GPS locations has shown that wolves selectively wait in ambush and kill beavers that forage farther from water. Wolf predation appears to be a consumptive and non‐consumptive force that constrains the distance beavers forage from water (Gable et al. 2023). However, there is a lack of documented direct observations of wolf predation on beavers and beavers' behavioral response to wolf predation. Consequently, although wolves regularly kill beavers in many systems, we know very little about how wolves kill beavers or how surviving beavers respond to the predation of group members.

One direct account of a wolf attacking and killing a beaver was a video recorded in October 2015 along a remote logging road in Quebec, Canada. To our knowledge, this was the only known video of this behavior published in the scientific literature at the time (Gable, Stanger, et al. 2018). This direct observation provided valuable insight into how wolves ambush beavers, how beavers defend themselves when attacked by wolves, and furnished evidence that wolves use higher‐order predation strategies and cognitive abilities when hunting beavers. Specifically, the wolf anticipated where the beaver traveled next and waited for the beaver to reach that area before launching an attack.

In September 2023, we captured a different video via a remote camera of a wolf killing a beaver who was foraging on a feeding trail. The remote camera also captured observations of beaver foraging on this trail before and after the predation event, yielding a rare data point on how predation may alter the foraging behavior of surviving group members. Given this observation, the objectives of this study were to: (1) describe in detail how the wolf in our observation caught and killed the beaver, (2) compare our observation to that recorded in 2015 (Gable, Stanger, et al. 2018) to identify similarities and/or differences; and (3) document the foraging behavior of beavers before and after the predation event.

## 2 Materials and Methods

### 2.1 Study Area

**FIGURE 1:**
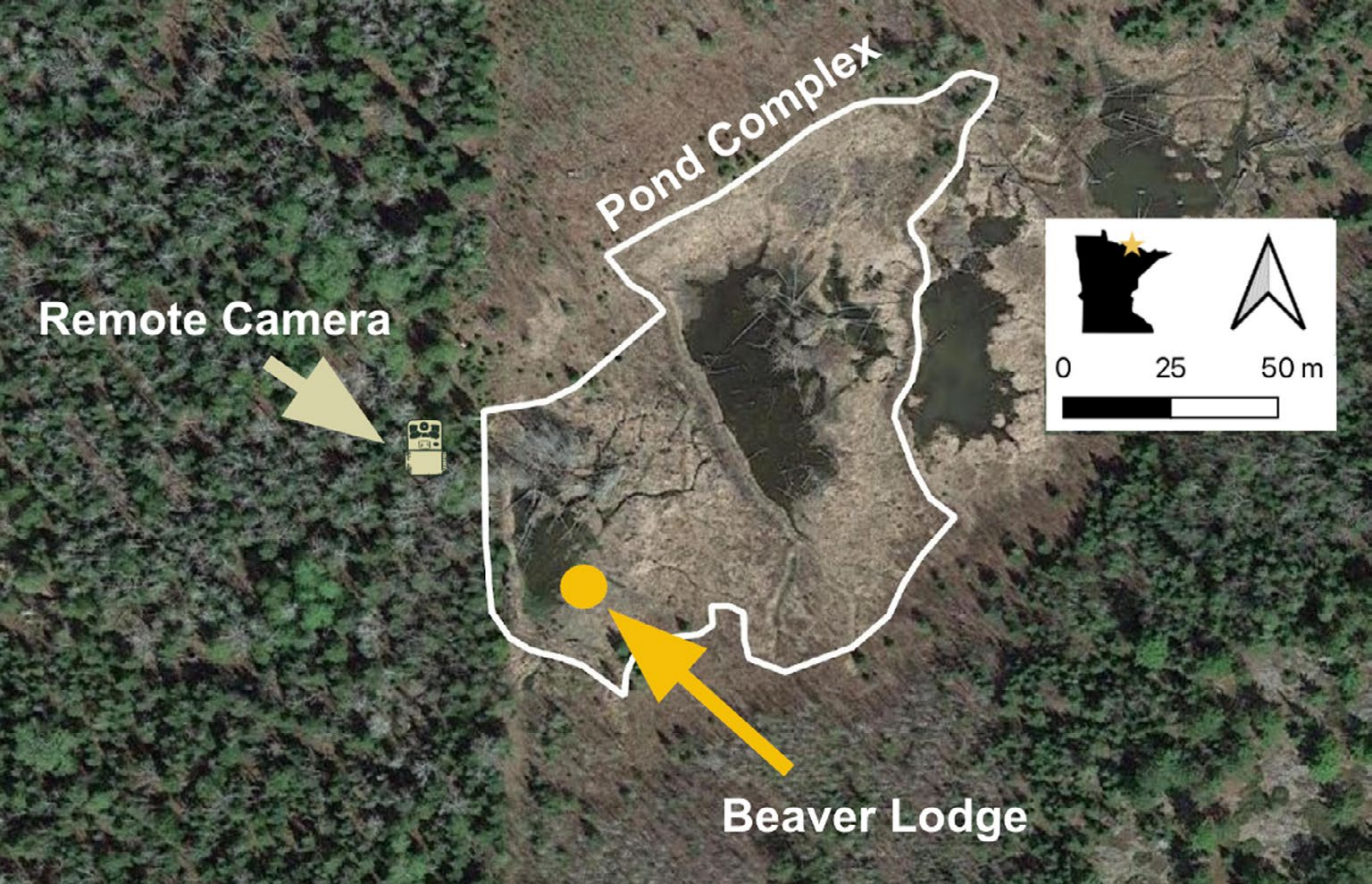
Map of pond complex in the Greater Voyageurs Ecosystem, Minnesota, USA where we recorded a video of a wolf killing a beaver with a remote camera on September 17th, 2023. Imagery from Google Earth Pro (2015).

Our observation occurred at a beaver pond complex (hereafter referred to as “pond”) south of Voyageurs National Park, in the Greater Voyageurs Ecosystem (GVE). For more information on the GVE, see Gable et al. (2020). We defined the pond complex as the pond where we placed the remote camera and the ponds immediately adjacent to that pond. We defined individual ponds as ponds separated by dams that spanned the entire pond and were visible on the most recent aerial imagery. The pond was approximately 100 m at its widest and 67 m at its longest and was bisected by a beaver dam. The beaver lodge was built on the shore of the south‐western edge of the pond (Figure 1). The area around the pond mainly consisted of dense deciduous forest made up of aspen (*Populus spp.*), alder (*Alnus spp.*), ash (*Fraxinus spp.*), elm (*Ulmaceae spp.*), birch (*Betulaceae spp.*), hazel (*Betulaceae spp.*), and maple (*Sapindales spp.*). When we began this study, the social group occupying the pond consisted of at least two adult beavers and one kit.

### 2.2 Remote Camera Deployment

We deployed a remote camera (Recon Force Elite HP5, Browning Trail Cameras, USA) on a 53‐m‐long feeding trail for 48 nights from September 7th to October 25th, 2023, as part of broader research to non‐invasively monitor beavers via hair snares made from barbed wire (Freund and Bump 2025). We programmed the camera to record a 20‐s video when activated. We set video quality to “ultra” (a resolution of 1080p with 60 frames per second), motion detection to “long range” (30.48 m), trigger speed to “fast” (0.1 s), infrared flash power mode to “long range” (39.62 m), smart infrared video to “off”, and SD management to “off”. We mounted the camera on the trunk of a balsam fir, with the bottom of the camera 50 cm off the ground. We pointed the camera down an active beaver feeding trail (parallel to the trail) toward the pond, 124° southeast. The hair snare (barbed wire) is visible in the video. When we collected hair from the hair snare (once every ~7 days; Freund and Bump 2025) we investigated the area around the camera for evidence of predation (e.g., blood, beaver or wolf hair, tracks, signs of struggle, and beaver remains). The camera and hair snare was deployed on land owned by the State of Minnesota, and permits are not required to deploy remote cameras on state‐owned land. However, we did have a permit from the Minnesota Department of Natural Resources for collecting hair samples from beavers via hair snares (permit no: 35003).

### 2.3 Remote Camera Video Analysis

After we collected the camera, we reviewed the footage and recorded the species of animal captured in each video, the time and date the video was recorded, the animal behavior captured (see metadata for description of behaviors), the age class of the animal captured (neonate or adult), and the number of individuals captured using QuickTime Player Version 10.5 (1150.4.1) following Freund and Bump (2025).

As part of the Voyageurs Wolf Project, we have studied wolves in the GVE since 2015 by fitting wolves with ear‐tags and GPS‐collars (Gable et al. 2016). This camera was deployed near the center of the Half‐Moon Pack territory, and the pack consisted of a breeding pair, two yearlings, and three pups during the period the camera was deployed. When we observed a wolf in footage captured by this camera, we determined if the individual was collared and/or ear‐tagged, and if so, recorded the ID on the ear‐tags when possible. Importantly, the breeding male of the Half‐Moon Pack wore a non‐functional GPS‐collar with green ear‐tags that had “V094” written on them at this time. The study followed Institutional Animal Care and Use Committee approvals by the U.S. National Park Service (MWR VOYA WINDELS WOLF) and the University of Minnesota (protocol ID: 1905‐37051A).

## 3 Results

### 3.1 The Predation Event

**FIGURE 2:**
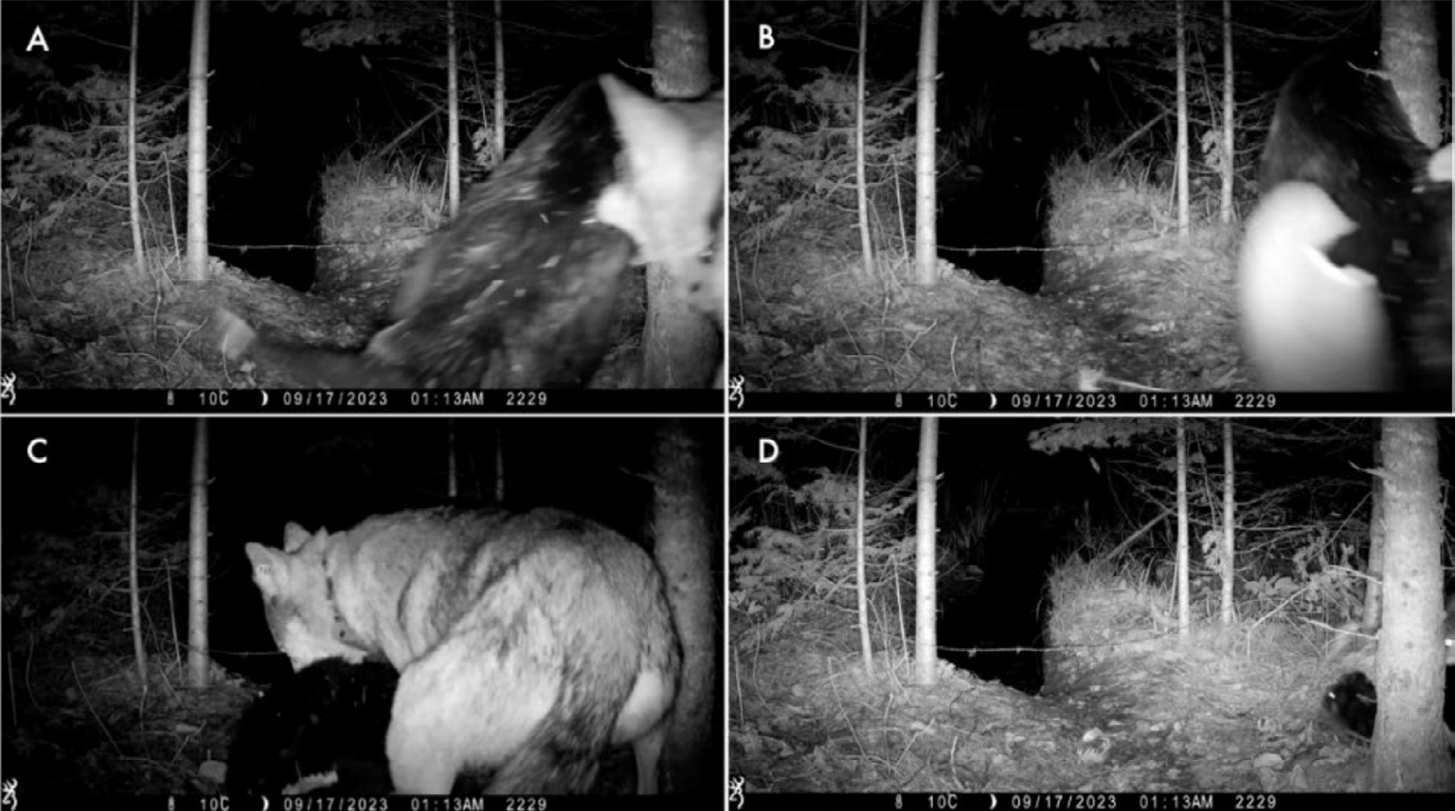
A video from a remote camera showing Wolf V094, a breeding male with a GPS collar, attacking a beaver on an active beaver feeding trail in the Greater Voyageurs Ecosystem, Minnesota, USA. Panel (A) shows V094 biting the beaver for the first time in the video; Panel (B) shows V094 pulling the beaver away from the water; Panel (C) shows V094 biting the beaver again after they got loose and ran towards the water; Panel (D) shows V094 pulling the beaver back away from the water for the final time recorded. The barbed wire was a hair snare used to collect beaver hair non‐invasively for another project (see Freund and Bump 2025).

At 1:13 a.m. on September 17th, we recorded a video of Wolf V094, the breeding male of the Half‐Moon Pack, attacking a beaver on land (Figure 2). We identified Wolf V094, who wore a non‐functional GPS‐collar, based on his ear‐tags being clearly visible in the video. The entire recorded interaction was 18 s. In the video, V094 grabbed the beaver by their right side in the center of their torso and pulled the beaver away from the water. Less than a second later, the beaver reappeared in the video, V094 lost his grip on the animal, and the beaver headed toward water. V094 then bit the top of the beaver's neck, and turned around, pulling the beaver back away from the water and out of the frame. Eleven seconds later, V094's tail and back leg entered and exited the frame. One second after that, the beaver entered the frame heading for the water. V094 then bit the beaver's back and pulled the beaver backward out of the video frame.

Later that day, at 9:11 pm, an uncollared wolf with a distended stomach walked across the feeding trail carrying a beaver skull with fur attached. The same uncollared wolf activated the camera again at 9:59 pm. We visited the site 2 days after the video was recorded to confirm that V094 killed the beaver. We found dried organs, blood, and tufts of beaver hair next to the camera, as well as beaver stomach contents 26 m from the water along the feeding trail.

### 3.2 Beaver Activity Before and After the Kill

**FIGURE 3:**
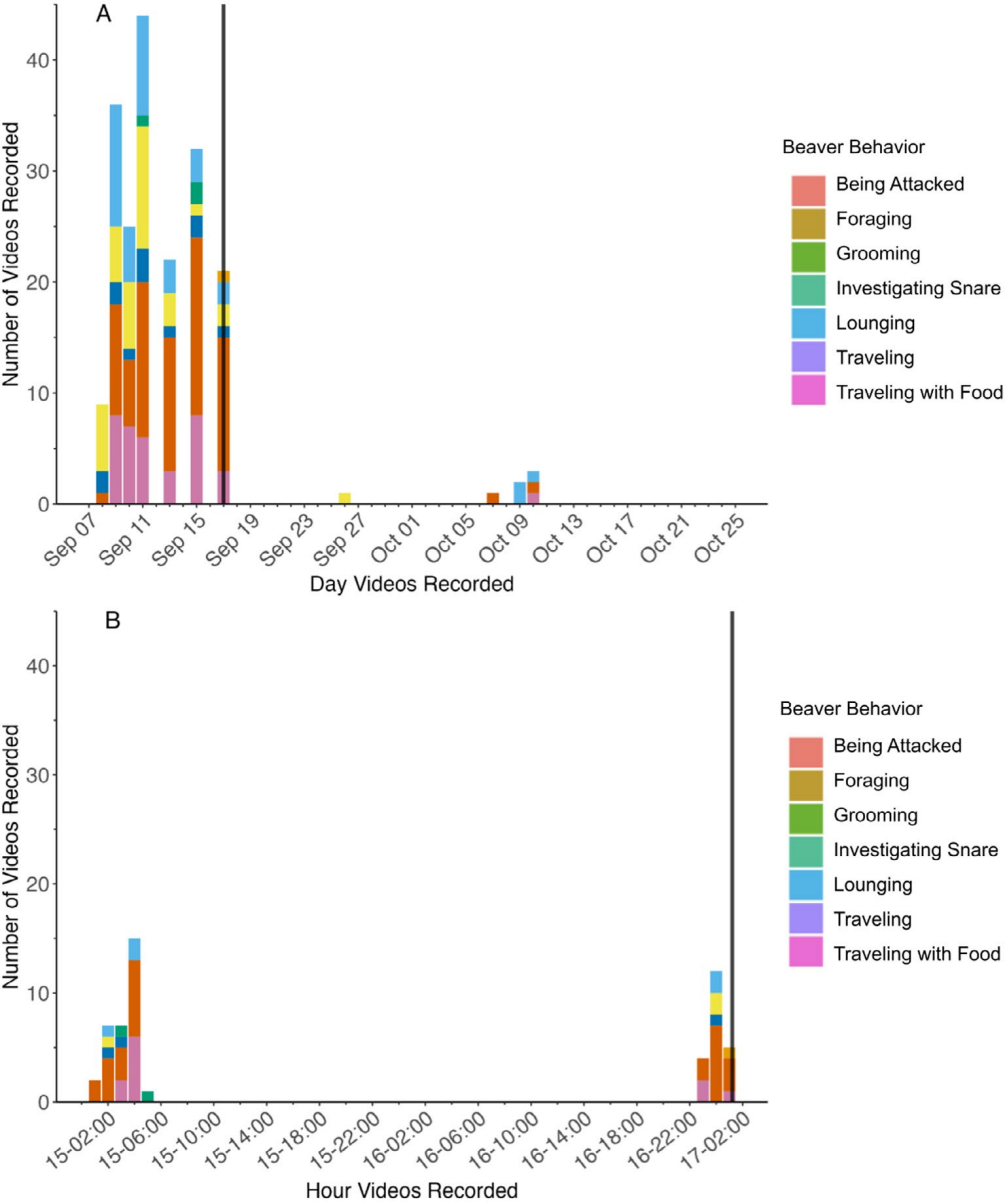
Number of 20‐s long videos of beavers captured via a remote camera placed on a beaver feeding trail in the Greater Voyageurs Ecosystem, Minnesota, USA. The remote camera was deployed from September 7th to October 25th 2023, but no videos of beavers were recorded after October 10th. The vertical black line indicates when wolf V094 killed a beaver in front of the remote camera on September 17th at 1:13 a.m. as indicated by the behavior classification “Being attacked”. “Investigating snare” indicates that the beaver in the video is sniffing or biting the snare. Panel (A) shows videos captured daily from September 7 to October 11 (month day). Panel (B) shows videos captured hourly from September 15 to 17 (day‐hour: minute) to better illustrate fine‐scale temporal changes in the behavior of beavers around the kill. Before the kill the camera recorded beavers at a rate of 17.6 beavers/day and after the kill the camera recorded beavers at a rate of 0.2 beavers/day.

Although our camera was deployed from September 7th to October 25th, beavers were only captured on the camera from September 7th to October 10th. No beavers were captured from October 10th to October 25th. Prior to the kill (September 7 to September 17, 11 days), our camera captured beavers foraging on the trail seven out of the 11 days (Figure 3), totaling 64.3 min of beaver activity observed by the remote camera. The camera specifically recorded beavers at a rate of 17.6 videos per day. We recorded a maximum of three beavers together at one time, and four videos where at least two beavers were on the trail at the same time in the video. From the size of the beavers recorded, we determined there were at least two adults and one kit in the family group before the wolf killed the beaver. After the kill (September 18 to October 10, 23 days), we captured beavers foraging on the trail 4 out of the 23 days, totaling 2.7 min of beaver activity observed by the remote camera. The camera specifically recorded beavers at a rate of 0.2 videos per day.

## 4 Discussion

### 4.1 Wolf Predation on Beavers: Observational Comparisons

The behavior of the wolf we observed aligns with the 2015 observation from Quebec (Gable, Stanger, et al. 2018). In both videos, wolves repeatedly bit and released the beaver and targeted the beavers' midsection, presumably to deliver lethal wounds while protecting themselves from the beavers' sharp teeth and powerful bite force. Additionally, our observation and the one in Quebec showed wolves dragging beavers away from water. This behavior suggests that wolves may be aware of beavers' increasing vulnerability farther from the water, or at a minimum that wolves may be aware that beavers could more readily escape if they reach water.

A major difference between the observation in Quebec and the one we documented is that the beaver killed in our observation was much larger. Larger beavers likely pose an increased risk to wolves than smaller beavers and are likely more difficult to kill. Adult beavers are often a similar weight (> 22 kg, Smith and Peterson 1991) or even larger than many adult wolves in the GVE (average wolf weight is 28 kg with female wolves commonly weighing~20–25 kg; Gable and Windels 2018). Thus, large adult beavers, given their size and strength, are more capable of fending off an attack compared to smaller beavers. Additionally, adult beavers have larger teeth than young beavers and likely a more powerful bite (Rosell and Campbell‐Palmer 2022). We do not currently know if wolves assess the cost–benefit of attacking beavers of certain sizes or if predation on beavers of any size by wolves is solely based on opportunity and circumstance. Notably, however, V094 was a large male (~38 kg) likely near the peak of his predatory abilities (6–7 years old) (MacNulty et al. 2009).

### 4.2 Can a Single Predation Event Alter Foraging Behavior?

After Wolf V094 killed the beaver, the surviving colony members scarcely used the trail for foraging. However, we cannot say with any certainty, given we have a sample size of one, that this change in behavior was driven by predation. Yet, the notion that such a change in beaver behavior was possibly due to a single predation event is intriguing: is it possible or even plausible to think wolves might immediately alter where or the extent to which beavers forage through predation? Our discussion below is intended to develop distinct possibilities that we think could be useful for future researchers who are studying and thinking about this predator–prey dynamic.

In response to the killing of a social group member, we think it possible that beavers may quickly adjust foraging patterns (e.g., foraging location and duration) in response to olfactory cues left from the predation event. Many studies have investigated how beaver's perceive predation risk using predator scents as proxies for the presence of predators (Engelhart and Müller‐Schwarze 1995; Rosell and Campbell‐Palmer 2022; Rosell and Sanda 2006; Salandre et al. 2017; Severud et al. 2011). When wolves kill beavers on feeding trails, multiple odorants are deposited, including odorants from the wolf or wolves, as well as those emanating from the beaver carcass. Given beavers' well‐developed olfactory abilities (Rosell and Campbell‐Palmer 2022), beavers may detect the high concentration of odorants from a recent predation event. For instance, based on our experience documenting > 700 wolf‐killed beavers to date, humans can readily smell the remains of freshly killed beavers from > 15 to 25 m away; beavers, which have much more developed olfactory systems, almost certainly could detect these odors much farther away. Importantly, we are not stating that wolf predation does indeed influence beaver foraging via this mechanism but rather that we think it is plausible, and future work should consider it.

Wolf predation likely also influences beaver foraging by immediately reducing the number of beavers in a social group. The average social group size for beavers in the GVE is ~5, and the death of one beaver, on average, is a 20% reduction in group size (Gable and Windels 2018). Wolves that kill a group member at a beaver pond would have an immediate impact on foraging activities because there are fewer beavers foraging. If every beaver forages equally, the loss of a social group member would reduce foraging by 20%. Wolves would not have such an effect if wolves killed a beaver that does little‐to‐no foraging (i.e., kits) or if group members increase foraging productivity to compensate for the lost group member. However, because adult beavers forage more than other group members (Rosell and Campbell‐Palmer 2022), wolves may have an outsized impact on the overall foraging of the social group when they kill adult members.

Given the cryptic nature of wolf predation on beavers, which primarily occurs in the dense forests of boreal ecosystems, our direct observation provides a unique data point. We propose a future direction of wolf‐beaver research that thoroughly investigates changes in foraging rates of beaver groups surrounding predation events by wolves. Methods to understand foraging changes may include placing remote cameras on all beaver feeding trails at ponds, regularly checking trails for predation events, and calculating any changes in trail use before and after the predation. A more experimental approach might be to examine how beavers alter foraging when odorants typically associated with a kill (e.g., blood, hair, stomach/intestinal contents from beaver carcasses) are placed on a feeding trail with a study design similar to Severud et al. (2011). Although such investigations would require intensive monitoring of ponds and social groups over long periods, the information gained would greatly increase our knowledge of how beavers respond to predation. Previous work on wolves hunting freshwater fish began with a single documented observation (Gable, Windels, et al. 2018), led to the collection of multiple years of intensively collected evidence, and eventually supported the idea that the behavior is likely widespread across similar ecosystems (Freund et al. 2023). We hope that our observation motivates others to explore intensive methods to determine what immediate effects, if any, wolf predation has on beaver behavior and beaver foraging specifically.

## Author Contributions

**Danielle R. Freund:** conceptualization (lead), data curation (lead), formal analysis (lead), investigation (lead), methodology (lead), writing – original draft (lead), writing – review and editing (lead). **Thomas D. Gable:** conceptualization (equal), writing – review and editing (equal). **Austin T. Homkes:** writing – review and editing (equal). **Olivia R. Jensen:** investigation (equal), writing – review and editing (supporting). **Sage Patchett:** investigation (equal), writing – review and editing (supporting). **Joseph K. Bump:** conceptualization (equal), funding acquisition (lead), investigation (supporting), methodology (supporting), project administration (lead), resources (lead), supervision (lead), writing – review and editing (supporting).

## Conflicts of Interest

The authors declare no conflicts of interest.

## Supporting information


Data S1:

Data S2:

Data S3:

## Data Availability

Remote camera data as well as associated code for the pond where the kill occurred are accessible as Supporting Information (Data [Link], [Link]).
