# Supplementary material for: Can Wolf Predation Immediately Alter the Foraging Behavior of Beavers?: Video of a Wolf Killing a Foraging Beaver
Source: Ecol Evol. 2025 May 10;15(5):e71357. doi: 10.1002/ece3.71357 (PMC12065075; doi:10.1002/ece3.71357)
Supplement: Supplementary file 2 — Data S2: [file ECE3-15-e71357-s001.pdf]

Can a single predation event alter foraging behavior?: Video of a wolf killing a foraging beaver

Freund et al.

Table of contents

Authors . . . 1
Background . . . 2
Loading Packages . . . 2
Cleaning Video Data . . . 2
General summary of camera deployment . . . 3
What animals were detected on the camera? Across what dates? . . . 3
How many times were beavers and wolves detected on the camera and at what time? . . . 3
Summarizing the number of beaver events before and after the predation event 7

Authors

Dani R. Freund(1,2), Thomas D. Gable(*1), Austin T. Homkes(1), Olivia R. Jensen(1), Sage Patchett(1), Joseph K. Bump(1)

*Corresponding author: thomasd.gable@gmail.com

(1)Department of Fisheries, Wildlife, and Conservation Biology, University of Minnesota, 2003, Upper Buford Circle, St Paul, MN 55108, USA

(2)Department of Environmental and Life Sciences, Trent University, Peterborough, ON, Canada, K9L 0G2

Background

We deployed a remote camera (Browning, Recon Force Elite HP5, Browning Trail Cameras, USA) on a 53-meter-long feeding trail for 49 days from September 7th to October 25th, 2023, as part of broader research to non-invasively monitor beavers via hair snares made from barbed wire (Freund & Bump, 2025). We programmed the camera to record a 20-second video (1920x1080p) when activated. We set video quality to "ultra" (60 frames per second), motion detection to "long range" (30.48 meters), trigger speed to "fast" (0.1 seconds), infrared flash power mode to "long range" (39.62 meters), smart infrared video to "off", and SD management to "off". We mounted the camera on the trunk of a balsam fir, with the bottom of the camera 500 cm off of the ground. We pointed the camera down an active beaver feeding trail (parallel) towards the pond, 124° southeast. The hair snare (barbed wire) is visible in the video. When we collected hair from the hair snare (once every ~7 days) we investigated the area around the camera for evidence of predation (e.g., blood, beaver or wolf hair, tracks, signs of struggle, and beaver remains). Human observers recorded the species of animal captured in each video, time and date video occurred, behavior (see supplementary material for description of behaviors), age class (neonate or adult), and number of individuals using QuickTime Player Version 10.5 (1150.4.1).

Loading Packages

```
library(googledrive) # for downloading google files
library(dplyr) # for organizing workflow
library(tidyr) # for organizing data
library(ggplot2) # for making graphs
library(ggridges) # for making ridge plots
```

Downloading Data

Cleaning Video Data

```
# converting dates, times, and integers
# combining time and date columns
snare_47 <- tidyr::unite(snare_47, date.recorded.time.start, date.video.recorded, time.24.
# converting date to POSIX format
snare_47$date.recorded.time.start<-as.POSIXct(snare_47$date.recorded.time.start, format =

# converting all numbers to integers
integer.cols <- c("degrees.C", "max.number.of.individuals", "min.number.of.individuals", "
```

```
snare_47[integer.cols] <- lapply(snare_47[integer.cols], as.integer)
```

General summary of camera deployment

```
# summarizing number of days camera took videos
snare_47 %>% dplyr::summarise(min = min(date.recorded.time.start),
                              max = max(date.recorded.time.start),
                              number.of.days.recording = (round(max-min)),0)
```

```
                  min                 max number.of.days.recording 0
1 2023-09-07 22:36:00 2023-10-25 12:18:00                  48 days 0
```

added 1 day to count the last day it was deployed

What animals were detected on the camera? Across what dates?

```
# summariizng number of each animal caught on camera
#ggplot(snare_47, aes(x=date.recorded.time.start)) + geom_histogram(aes(fill=main.species)

# summarizing animals caught on camera and on what days
#ggplot(snare_47, aes(x = date.recorded.time.start, y = main.species, fill = main.species)

# average number of beavers recorded per video
snare_47 %>% filter(main.species == "beaver") %>% summarize(average.beavers.per.video = me
```

```
  average.beavers.per.video
1                  1.079208
```

How many times were beavers and wolves detected on the camera and at what time?

```
# subsetting any videos with a wolf or beaver in them
beavers_wolves <- subset(snare_47, snare_47$main.species == "beaver"|
                         snare_47$main.species == "wolf"|
                         snare_47$secondary.species == "beaver")

# accounting for beavers as secondary species (in this case, we do not care about the prim
beavers_wolves$adjusted.species <- ifelse(beavers_wolves$secondary.species == "beaver", "b
```

```
beavers_wolves$adjusted.behavior <- ifelse(beavers_wolves$secondary.species == "beaver", b

# replacing traveling with food with foraging
beavers_wolves$adjusted.behavior <- ifelse(
  beavers_wolves$adjusted.species == "beaver" &
    beavers_wolves$adjusted.behavior == "travelingwithfood",
  "foraging", beavers_wolves$adjusted.behavior)

# graph by day
beavers_wolves$date_notime <- as.Date(format(as.Date(beavers_wolves$date.recorded.time.sta
all_dates <- seq(as.Date("2023-09-07"), as.Date("2023-10-26"), by = "1 day")
all_dates <- as.Date(all_dates, "%Y-%m-%d")
kill <- as.Date(as.Date("2023-09-17"), "%Y-%m-%d")

p1 <- beavers_wolves %>%
  filter(main.species == "beaver" & main.behavior != "swimming") %>%
  ggplot(aes(x = date_notime, fill = main.behavior)) +
  geom_bar() +
  geom_vline(xintercept = kill, linewidth = 1.25, alpha = 0.75) +
  labs(x = "Day Videos Recorded", y = "Number of Videos Recorded",
       fill = "Beaver Behavior") +
  theme_classic() +
  theme(
    axis.title.x = element_text(size = 15),
    axis.title.y = element_text(size = 15),
    axis.text.x = element_text(size = 15, angle = 45, hjust = 1),
    axis.text.y = element_text(size = 15),
    legend.text = element_text(size = 15),
    legend.title = element_text(size = 15)
  ) +
  scale_x_date(
    limits = c(
      min(all_dates),
      as.Date("2023-10-25") # Extend to October 25th
    ),
    breaks = all_dates[seq(1, length(all_dates), by = 4)],
    minor_breaks = seq(min(all_dates), as.Date("2023-10-25"), by = 1), # Minor ticks every
    date_labels = "%b %d",
    guide = guide_axis(minor.ticks = TRUE) # Enable minor ticks
  ) +
  scale_y_continuous(
```

```
    limits = c(0, 45),
    expand = c(0, 0),
    minor_breaks = waiver(), # Default minor ticks for y-axis
    guide = guide_axis(minor.ticks = TRUE) # Enable minor ticks
  )

colorblind_friendly_colors <- c(
  "#E69F00", "#56B4E9", "#009E73", "#F0E442",
  "#0072B2", "#D55E00", "#CC79A7"
)

p1 <- p1 +
  scale_fill_manual(values = colorblind_friendly_colors) +
  annotate("text", x = min(all_dates) + 0.5,
           y = max(45),
           label = "A", size = 6, hjust = 0, vjust = 1) +
  coord_cartesian(clip = 'off')

# graph by hour that the events occurred
library(scales)

beavers_wolves$hour <- as.Date(format(as.Date(beavers_wolves$date.recorded.time.start, "%Y
start <- as.Date("2023-09-15", "%Y-%m-%d")
end <- as.Date("2023-09-19", "%Y-%m-%d")
kill.hour <- as.POSIXct("2023-09-17 01:13:00", format = "%Y-%m-%d %H:%M:%S")
all_dates_short <- seq(as.POSIXct("2023-09-15 00:00:00"), as.POSIXct("2023-09-19 23:00:00"

beavers_wolves_filtered <- beavers_wolves %>%
  filter(date_notime >= start & date_notime <= end)

p2 <- beavers_wolves_filtered %>%
  filter(main.species == "beaver" & main.behavior != "swimming") %>%
  mutate(date_and_hour = format(date.recorded.time.start, "%Y-%m-%d %H:00:00")) %>%
  ggplot(aes(x = as.POSIXct(date_and_hour), fill = main.behavior)) +
  geom_bar() +
  labs(x = "Hour Videos Recorded", y = "Number of Videos Recorded",
       fill = "Beaver Behavior") +
  theme_classic() +
  theme(
    axis.title.x = element_text(size = 15),
    axis.title.y = element_text(size = 15),
```

```
    axis.text.x = element_text(size = 15, angle = 38, hjust = 1),
    axis.text.y = element_text(size = 15),
    legend.text = element_text(size = 15), # Increase legend item size
    legend.title = element_text(size = 15) # Increase legend title size
  ) +
  geom_vline(xintercept = kill.hour, linewidth = 1.25, alpha = 0.75) +
  scale_x_datetime(
    breaks = date_breaks("4 hour"),
    date_labels = "%d-%H:%M",
    guide = guide_axis(minor.ticks = TRUE)
  ) +
  scale_y_continuous(
    limits = c(0, 45), # Set y-axis to range from 0 to 45
    expand = c(0, 0),
    guide = guide_axis(minor.ticks = TRUE)
  )

p2 <- p2 +
  scale_fill_manual(values = colorblind_friendly_colors) +
  annotate("text",
           x = as.POSIXct(min(beavers_wolves_filtered$date.recorded.time.start)) + 60, # S
           y = 45,  # Position near the top of the y-axis
           label = "B",
           size = 6,
           hjust = 0,
           vjust = 1) +
  coord_cartesian(clip = 'off')

# COMBINING GRAPHS
library(ggpubr)
arranged_plots <- ggarrange(p1, p2, ncol = 1, common.legend = FALSE, legend = "right")
ggsave("/Users/danifreund/Desktop/Research/wolf_attacking_beaver_video/wolf_attacking_beav
# date and time kill happened
kill_date <- as.POSIXct("2023-09-17 01:13:00")

#ggplot(beavers_wolves, aes(x = date.recorded.time.start, color = adjusted.behavior, linet
  #geom_vline(xintercept = (kill_date), color = "red") +
  #theme_classic()
```

There were no BHA's or beaverkills recorded at this spot this year from the collared wolves

Summarizing the number of beaver events before and after the predation event

```
# before the predation event
before_kill<-beavers_wolves %>% subset(main.species == "beaver") %>%
                              subset(date.recorded.time.start < kill_date)
before_kill_count <- before_kill %>% group_by(date_notime) %>% summarize(n=n())
# 11 nights between September 7 and 17th
sum(before_kill_count$n)/11
```

```
[1] 17.54545
```

```
# summing minutes of beavers caught before kill (each video was 20 seconds)
(((sum(before_kill_count$n)*20)/60))
```

```
[1] 64.33333
```

```
# finding the average amount of time between videos
before_kill$date.recorded.time.start <- as.POSIXct(before_kill$date.recorded.time.start, f

# Compute time differences in seconds
time_diffs <- diff(before_kill$date.recorded.time.start)
print(time_diffs)
```

```
Time differences in mins
  [1]     2   137     8   117     5     2     2     1     4  1126     2     2
 [13]     5    26     3     1     3     9    23     2     6     2     7     5
 [25]    22    45    10     4     8    38     2     2     2     1    37     2
 [37]     1     3     4     5     4     3     5     2     8     3  1128     2
 [49]     9     6    31     1    41     1     6     4     2     2     4     6
 [61]     5     3     1     2     4     2     1     2     9     3     9   248
 [73]   136  1080     1     2    20     2     1    61     2     2     1     2
 [85]     2     4     4     3    10    77     3     5     4     4     5     4
 [97]     5     2     1     1     2     2     6     2     2     4     8     3
[109]     1     6     1     2     2     1     1     6     2  2463     2     4
[121]     5     2     2     3     3     3     5     7     2     1     2     3
[133]    10     5    27     1     2    16     1   234  2728     2    20     2
[145]     1    21     9     1     5    24     2    21     8     4     4     4
[157]    10     2     4     5     5     7     1     5     3     5     3     5
[169]     5     4    57  2511     5     2     3     9    10     2     1     5
[181]     2    10     1     2    14     4     3     2     1     5     3 -2679
```

```r
sd(time_diffs)/60
```

```
[1] 6.641087
```

```r
# Calculate the average time difference in minutes
avg_time_diff_minutes <- mean(time_diffs, na.rm = TRUE) / 60

# Print result
print(avg_time_diff_minutes)
```

```
Time difference of 0.9057292 mins
```

```r
# after the predation event
after_kill<-beavers_wolves %>% subset(main.species == "beaver") %>%
                          subset(date.recorded.time.start > kill_date)
after_kill_count <- after_kill %>% group_by(date_notime) %>% summarize(n=n())
# 23 nights between Sept 18 and October 10th
sum(after_kill_count$n)/23
```

```
[1] 0.3478261
```

```r
# 37 nights between Sept 18 and October 25th
sum(after_kill_count$n)/37
```

```
[1] 0.2162162
```

```r
# summing minutes of beavers caught after kill (each video was 20 seconds)
(((sum(after_kill_count$n)*20)/60))
```

```
[1] 2.666667
```

```r
# finding the average amount of time between videos
after_kill$date.recorded.time.start <- as.POSIXct(after_kill$date.recorded.time.start, for

# Compute time differences in seconds
```

```r
time_diffs <- diff(after_kill$date.recorded.time.start)
print(time_diffs)
```

```
Time differences in mins
[1] 15528    43  3369     2   870   299     5
```

```r
# Calculate the average time difference in minutes
avg_time_diff_minutes <- mean(time_diffs, na.rm = TRUE) / 60

# Print result
print(avg_time_diff_minutes)
```

```
Time difference of 47.89524 mins
```

```r
sd(time_diffs)/60
```

```
[1] 95.13866
```

```r
# percent decline in minutes beavers caught on video
(((((sum(before_kill_count$n)*20)/60))-(((sum(after_kill_count$n)*20)/60)))/(((sum(before_
```

```
[1] 95.85492
```

Determining number of beavers captured on average per video

```r
beavers_wolves %>% filter(main.species == "beaver" |
                            secondary.species == "beaver") %>%
  group_by(max.number.of.individuals) %>% summarize(n=n())
```

```
# A tibble: 3 x 2
  max.number.of.individuals     n
                      <int> <int>
1                         1   187
2                         2    18
3                         3     1
```
