# Supplementary material for: Can Wolf Predation Immediately Alter the Foraging Behavior of Beavers?: Video of a Wolf Killing a Foraging Beaver
Source: Ecol Evol. 2025 May 10;15(5):e71357. doi: 10.1002/ece3.71357 (PMC12065075; doi:10.1002/ece3.71357)
Supplement: Supplementary file 3 — Data S3: [file ECE3-15-e71357-s002.docx]

# ***Can wolf predation immediately alter the foraging behavior of beavers?: Video of a wolf killing a foraging beaver***

Danielle R. Freund^1,2^, Thomas D. Gable^*1^, Austin T. Homkes^1^, Olivia R. Jensen^1^, Sage Patchett^1^, Joseph K. Bump^1^

^*^Corresponding author: thomasd.gable@gmail.com

^1^Department of Fisheries, Wildlife, and Conservation Biology, University of Minnesota, 2003, Upper Buford Circle, St Paul, MN 55108, USA

^2^Department of Environmental and Life Sciences, Trent University, Peterborough, ON, Canada, K9L 0G2

**camera_video_of_a_wolf_killing_a_foraging_beaver**

Each line of data is an individual video of an animal recorded on a remote camera placed at a hair snare set at a beaver pond in the Greater Voyageurs Ecosystem in northern MN, USA.

- **hair.snare.ID:** ID’s of individual hair snares set around active beaver ponds to non-invasively collect beaver hair. Assigned numerically in order of deployment.
- **date.sd.card.collected:** MM-DD-YYY. Date an sd card was collected from hair snare. When sd cards were collected, we also checked snares for hair samples and replaced batteries in the cameras if the batteries were <50%. If there was hair on the snare, it was collected and recorded.
- **date.video.recorded**: MM-DD-YYYY. Date the video was recorded on the remote camera.
- **time.video.starts**: HH:MM in 12-hour format. Time the video began recording.
- **AM.PM:** If the “time.video.starts” was recorded in AM or PM.
- **time.24.hour:** HH:MM:SS in 24-hour format. Time the video began recording.
- **degrees.C:** Outside temperature at hair snare location. Recorded by remote camera.
- **main.species**: Common name of species that takes up the majority of the time of the video. Plural for species name was never used, even if there is was than one. If species could not be identified down to species, "unk" was put before common species name (such as unkweasel).
- **secondary.species:** Common name of second species that takes up less time of the video than the main species. If there is a third species in the video, it was written in the comments section.
- **max.number.of.individuals:** Maximum possible number of individuals in the video, regardless of species.
- **min.number.of.individuals:** Minimum possible number of individuals in the video, regardless of species. The only time max number of individuals and min number of individuals was recorded as different was if individuals left the video frame and then came back. In this case, unless we could identify them as the same individual from markings or individual characteristics, we recorded them.
- **max.number.of.adults:** Maximum number of known adults in the video.
- **max.number.of.neonates:** The max number of known neonates (kit,fawn,cub ect.) in the video. Yearlings were considered adults.
- **max.number.of.females:** The max number of known females in the video.
- **max.number.of.males:** The max number of known males in the video.
- **max.number.of.unk.sex:** The max number of individuals with unknown sex in the video.
- **main.behavior:** behavior that takes up the majority of the time of the video.
- **secondary.behavior:** behavior of secondary.species or if the main.species had another behavior that takes about approximately half of the video time.

Ethogram of behaviors entered to main.behavior and secondary.behavior column.

| traveling | animal(s) appears to be traveling USE TWO L'S | | | | | | | |
| --- | --- | --- | --- | --- | --- | --- | --- | --- |
| travelingwithfood | animal(s) are traveling with food in their mouth (e.g., fox with muskrat in mouth or wolf with deer leg) | | | | | | | |
| scentmarking | animal(s) appears to be scent-marking territory | | | | | | | |
| scentrolling | animal(s) appears to be rolling on the ground in an interesting scent | | | | | | | |
| scentrubbing | animal(s) are rubbing body (but not rolling on ground) on tree, bait, or some other object; very similar to scent-rolling | | | | | | | |
| foraging | animal(s) is feeding on vegetation or other food source | | | | | | | |
| drinking | animal(s) is drinking from water source | | | | | | | |
| urinating | animal(s) is urinating; animal(s) cannot be scent-marking (typically only applies to neonates and prey species) | | | | | | | |
| defecating | animal(s) is defecating | | | | | | | |
| social | animal(s) is interacting with conspecifics. | | | | | | | |
| interspecific | animal(s) is interacting with other species | | | | | | | |
| investigating | animal(s) appears to be checking out area in front of camera, this includes sniffing | | | | | | | |
| investigatingcamera | animal(s) is checking out camera/camera set-up | | | | | | | |
| swimming | animal(s) are swimming across a body of water. Animal must be floating to be considered swimming | | | | | | | |
| swimmingwithfood | animal(s) is exhibiting swimming behavior and clearly has food in its mouth (e.g., otter with crawyfish in its mouth) | | | | | | | |
| flying | animal(s) are flying across the screen (i.e., birds) | | | | | | | |
| hunting | animal(s) is actively hunting a prey species (note this is different than chasing; the predator must make an attempt at the prey species) | | | | | | | |
| fleeingpredator | animal(s) are fleeing a predator. Predator must be visible in pictures or videos within series. | | | | | | | |
| chasingprey | animal(s) are chasing a prey animal. Prey animal must be present in pictures or videos within series. | | | | | | | |
| fishing | animal(s) are catching fish or waiting near/in body of water to catch fish | | | | | | | |
| eatingberries | animal(s) is eating berries; this applies to all species (i.e. deer, bears, wolves, etc) | | | | | |  |  |
| eatingdeer | animal(s) is eating deer; it must not be bait | | | | | | | |
| eatingfish | animal(s) is eating fish | | | | | | | |
| eatingunk | animal(s) is eating prey item but species of prey is unknown; | | | | | | | |
| chewingbone | animal(s) is chewing bone; unclear what species the bone belongs to; animal(s) cannot be eating meat off the bone | | | | | | | |
| sleeping | animal(s) is sleeping | | | | | | | |
| lounging | animal(s) is sitting, wandering, hanging out in front of camera; animal(s) cannot be exhibiting any other behavior | | | | | | | |
| standing | animal(s) is standing without moving around. | | | | | | | |
| displaying | animal(s) is displaying (i.e. grouse drumming) | | | | | | | |
| climbing | animal(s) is climbing a tree; this must be the predominant behavior taking place (i.e. they should be in a tree for most of the series) | | | | | | | |
| begging | animal(s) is whining to beg for food at a den site. | | | | | | | |
| enteringexitingden | animal(s) is entering or exiting a known den. | | | | | | | |
| howling | animal(s) is howling. | | | | | | | |
| playing | animal(s) is interacting playfully with conspecifics or with a toy. | | | | | | | |
| inden | animal(s) is in den; not entering/exiting, playing, or any other behavior. | | | | | | | |
| rearingpups | adult wolf (or wolves) are caring for/interacting with pups; cannot including feeding behaviors' | | | | | | | |
| nursing | neonate(s) is nursing; cannot be eating hard food | | | | | | | |
| defendingden | adult wolf (or wolves) is defending the den from other animal(s) | | | | | | | |
| eatingbait | animal(s) is eating bait. Must clearly be a carcass left in front of the camera as bait. | | | | | | | |
| examiningbait | animal(s) is investigating bait; can be scent or carcass. Animal(s) must not be eating bait. | | | | | | | |
| unk | it is unclear what the animal(s) is doing; does not fit under any other category. | | | | | | | |
| relocatingpups | wolf(ves) moves pup from one den to another | | | | | | | |
| dominancesubmission | when 2 animals are engaged in behavior where one dominates the other animal into submission | | | | | | | |
| spawning | fish are spawning | | | | | | | |
| dabbling | waterfowl are dunking head into water to feed | | | | | | | |
| traprelated | animal(s) behavior is associated with traps (i.e. animal is caught in trap) | | | | | | | |
| dammaintenance | beaver(s) are mainting dam (i.e. adding mud, sticks, etc.) | | | | | | | |
| preening | bird(s) is preening (ie. any waterfowl,songbirds,etc) | | | | | | | |
| bathing | animal(s) is cleaning itself in a body of water; cannot be associated with fishing or drinking behaviors | | | | | | | |
| mating | animals are participating in mating behavior | | | | | | | |
| grooming | animal(s) is cleaning itself or another conspecific; this cannot be bathing | | | | | | | |
| caching | animal(s) is hiding/storing food (vegetation ex. pine cone or prey item) in a cache | | | | | | | |
| climbingwithfood | animal(s) are climbing with food in their mouth (e.g., red squirrel with a pine cone in their mouth) | | | | | | | |
| vocalizing | animal(s) is making vocal noises other than howling | | | | | | | |
| scratching | animal(s) is scratching an itch | | | | | | | |
| investigatingsnare | animal(s) is checking out the snare such as sniffing or biting the snare | | | | | | | |
| attacking | animal(s) are attacking another animal | | | | | | | |
| beingattacked | animal(s) are being attacked by another animal | | | | | | | |

- **wolf.ID:** If there is a wolf in the video that is a known individual from ear tags or markings, this is their ID that was previously assigned.
